# Genome-wide screen reveals important roles for ESCRT proteins in drug/ion resistance of fission yeast

**DOI:** 10.1371/journal.pone.0198516

**Published:** 2018-06-01

**Authors:** Yikun Yang, Qiannan Liu, Guanglie Jiang, Si Chen, Lina Zhou, Norihiro Sakamoto, Takayoshi Kuno, Yue Fang, Fan Yao

**Affiliations:** 1 Department of Microbial and Biochemical Pharmacy, School of Pharmacy, China Medical University, Shenyang, China; 2 Division of Food and Drug Evaluation Science, Department of Social/Community Medicine and Health Science, Kobe University Graduate School of Medicine, Kobe, Japan; 3 Department of Breast Surgery and Surgical Oncology, Research Unit of General Surgery, The First Affiliated Hospital of China Medical University, Shenyang, China; JAPAN

## Abstract

To study sodium homeostasis, we performed a genome-wide screen for deletion strains that show resistance to NaCl. We identified 34 NaCl-resistant strains. Among them, the largest group that consists of 10 genes related to membrane trafficking and 7 out of 10 genes are ESCRT proteins which are involved in cargo transportation into luminal vesicles within the multivesicular body. All of the ESCRT related mutants which showed sodium resistance also showed defects in vacuole fusion. To further understand the role of the ESCRT pathway in various ion homeostasis, we examined sensitivity of these ESCRT mutants to various cation salts other than NaCl, including KCl, LiCl, CaCl_2_, CoCl_2_, MgCl_2_, NiSO_4_ and MnCl_2_. While these ESCRT mutants showed resistance to LiCl, CoCl_2_ and MgCl_2_, they showed sensitivity to KCl, CaCl_2_, NiSO_4_ and MnCl_2_. Then we examined sensitivity of these ESCRT mutants to various drugs which are known to inhibit the growth of fission yeast cells. While these ESCRT mutants were more or equally sensitive to most of the drugs tested as compared to the wild-type cells, they showed resistance to some drugs such as tamoxifen, fluorouracil and amiodarone. These results suggest that the ESCRT pathway plays important roles in drug/ion resistance of fission yeast.

## Introduction

Sodium ion homeostasis is a vital cellular function ranging from prokaryotes to eukaryotes. In human, genetic defects in sodium ion homeostasis contribute to the risk of high blood pressure [1]. In agriculture, salt stress is an environmental pressure to crop plants, and genetic engineering of proteins that are involved in sodium ion homeostasis may lead to their increased salt tolerance [2, 3]. Thus, understanding sodium ion homeostasis has important implications for a wide range of fields including medicine [4], microbiology [5] and agriculture [6].

Construction of fission yeast *Schizosaccharomyces pombe* (*S*. *pombe*) deletion library has enabled us to perform genome-wide screen of mutants defective in various biological processes. Genome wide screen of sensitive and/or resistant deletion mutants has been performed for cadmium [7], cobalt [8], caffeine [9], immunosuppressant FK506 (tacrolimus) [10], antifungal drugs [11, 12], anticancer drugs [13, 14], and valproic acid [15].

In the present study, to study sodium homeostasis, we systematically screened fission yeast nonessential knockout library, and identified 34 NaCl-resistant strains. Among them, 7 genes are related to ESCRT (endosomal sorting complex required for transport) proteins, which are involved in cargo transportation into luminal vesicles within the multivesicular body. These ESCRT mutants also showed resistance to LiCl, CoCl_2_ and MgCl_2_, however, they showed sensitivity to other cation salts tested, such as KCl, CaCl_2_, NiSO_4_ and MnCl_2_. Likewise, these ESCRT mutants showed resistance to some drugs such as tamoxifen, fluorouracil (5-FU) and amiodarone, while they were equally or more sensitive to other drugs tested. These results suggest that the ESCRT pathway plays important roles in drug/ion resistance of fission yeast.

## Materials and methods

### *S*. *pombe* nonessential gene knockout library

*S*. *pombe* Haploid Deletion Mutant Library (Set ver 2.0) constructed by Bioneer Corporation and Korea Research Institute of Biotechnology and Bioscience (http://pombe.bioneer.co.kr/) were used in this study. These deletion strains were generated with a genetic background of *h*^+^ *leu1-32 ura4-D18 ade6-M210* or *-M216* using PCR-based deletion method [16]. The haploid deletion library used in this study consists of 3004 nonessential genes, each of which carries a defined deletion of a characterized or a putative nonessential open reading frame (ORF) replaced with the *kanMX4* cassette. Deletion of the target ORF was screened by G418 antibiotic selection.

### Media, genetic and molecular biology methods

The complete medium YPD (yeast extract-peptone-dextrose) and the minimal medium EMM (Edinburgh minimal medium) have been described previously [17]. YPD plates are supplemented with 225 mg/l adenine to produce YPDA (yeast peptone dextrose adenine) plates. Gene disruptions are abbreviated by the gene preceded by Δ (for example, Δ*vps25*). Proteins are denoted by Roman letters and only the first letter is capitalized (for example, Vps25) [18].

### Genome-wide screen for sodium chloride-resistant deletion mutants

We used streak assay for a preliminary screen and dilution-series spot assay for a secondary screen as reported previously [11–13]. In the preliminary screen, the log-phase cells were streaked onto YPDA or YPDA plus various concentrations of NaCl to screen sodium chloride resistant strain. Deletion cells that exhibited enhanced resistance in the preliminary screen were selected to carry out the secondary screen. To group the extent of the resistance, the deletion cells were spotted onto YPDA or YPDA containing 250mM NaCl. Three independent spot assays were done. All chemicals and reagents were purchased from commercial sources.

### Calcineurin-dependent response element (CDRE)—dependent reporter assay

Real-time monitoring of calcineurin-dependent response element (CDRE) reporter activity using the firefly luciferase reporter assay was performed as described previously [19]. To assess the effect of ESCRT mutations on CDRE activity, the multicopy reporter plasmid pKB5723 (3×CDRE::luc(2.2)) was transformed into wild-type and ESCRT mutant cells, and treated with 100mM CaCl_2_ or 100mM NaCl. D-luciferin sodium salt monohydrate (50 mM in sterile water as stock solution; Biosynth Corporation, Switzerland) was used as a substrate for firefly luciferase and was added to the cell suspension at 1:100 dilution. Light emission levels expressed as relative light units were measured at 1-min intervals for three hours using a luminometer (AB-2350; ATTO Co., Tokyo, Japan) at 27°C.

### Miscellaneous methods

Methods in light microscopy, such as fluorescence microscopy and differential interference contrast (DIC) microscopy were performed as described previously [20, 21] by using a Nikon Eclipse Ni-U microscope equipped with a DS-Qi2 camera (Nikon Instruments Inc., Japan). Database searches were performed using the National Center for Biotechnology Information BLAST network service and the fission yeast *S*. *pombe* database search service (http://www.pombase.org/).

## Results

### Identification of NaCl-resistant mutants

**Fig 1:**
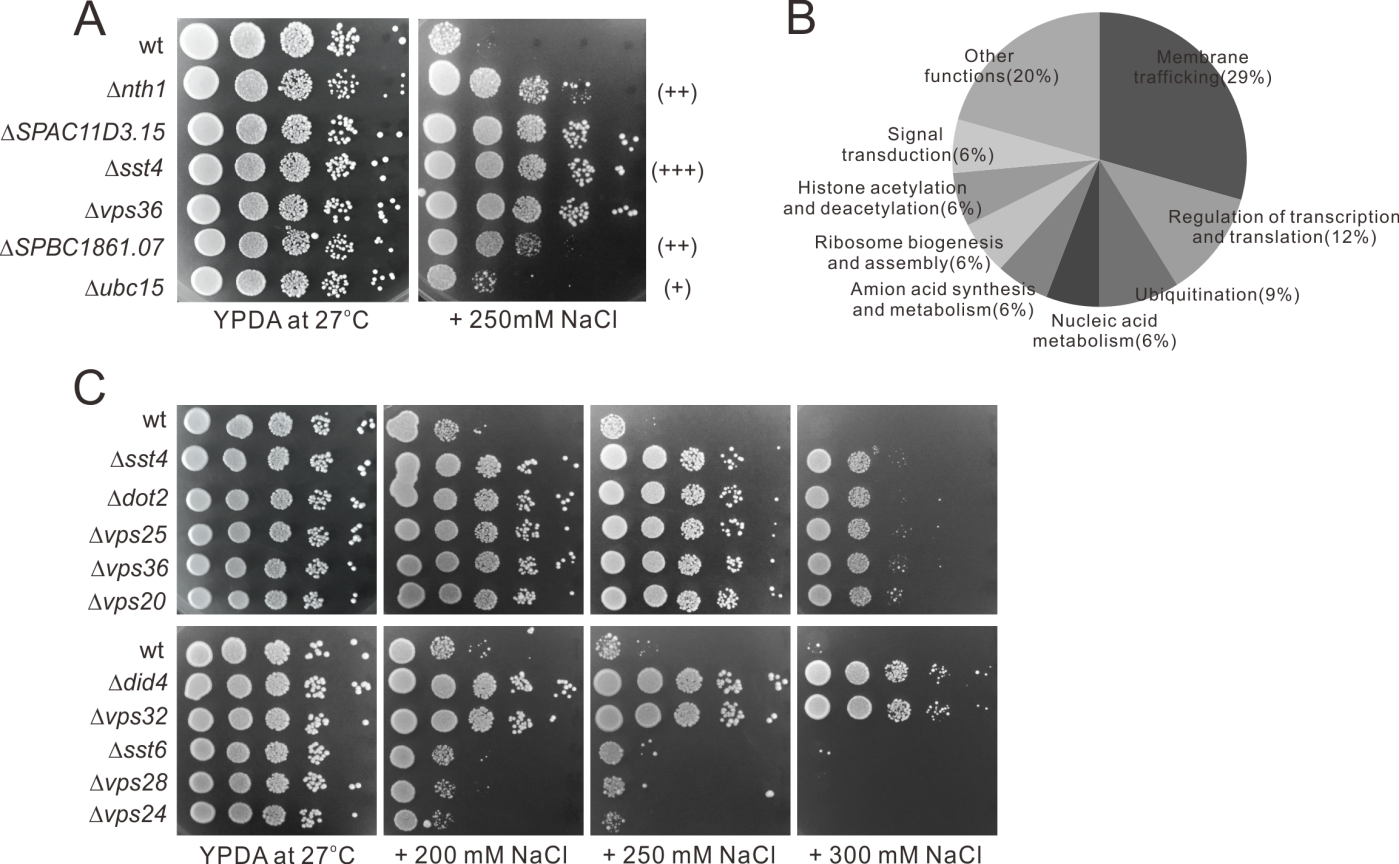
Representative examples of the *S*. *pombe* deletion mutants screened for NaCl resistance. (A) Representative examples of the NaCl-resistant mutants. Wild-type (wt) cells and deletion mutant cells grown at log phase were spotted onto each plate as indicated and then incubated at 27°C for 4 days. (B) Distribution of functional categories for the 34 NaCl-resistant genes in fission yeast. (C) Most of the ESCRT mutants showed resistance to sodium chloride. Wild-type (wt) cells and ESCRT mutant cells grown at log phase were spotted onto each plate as indicated and then incubated at 27°C for 4 days.

To identify nonessential genes associated with increased resistance to sodium chloride, we performed a genome-wide screen and isolated 34 deletion strains that displayed varying levels of resistance to NaCl. The resistance to NaCl was scored as follows: strong resistance (+++) indicating that the deletion cells grow very well on the plates containing 250 mM NaCl and the third and fourth spot could be observed (Fig 1A), moderate resistance (++) indicating that the second spot could be observed (Fig 1A), and mild resistance (+) indicating that the first spot could be observed but the colony sizes of the deletion mutants were mostly larger than those of the wild-type cells on the plates containing 250 mM NaCl (Fig 1A). Among the 34 NaCl-resistant mutants, 16 mutants were strongly resistant (+++), 10 mutants were moderately resistant (++), and 8 mutants were mildly resistant (+) (Table 1). The 34 genes were grouped by their functions (Table 1 and Fig 1B). The largest group consisted of genes involved in membrane trafficking (10/34 = 29.4%), the second and third largest groups consisted of genes involved in the regulation of transcription and translation (4/34 = 11.8%) and ubiquitination (3/34 = 8.8%), respectively. Other groups consisted of genes involved in nucleic acid metabolism, amino acid synthesis and metabolism, ribosome biogenesis and assembly, histone acetylation and deacetylation, signal transduction and there were also a variety of genes with other known functions in the biological system (Table 1 and Fig 1B).

**Table 1:** *S*. *pombe* genes identified in NaCl -resistant screen.

Category and systematic gene name	Common gene name	Gene description	NaCl resistance
**Membrane trafficking**			
SPAC19A8.05c	*sst4*	ESCRT 0 complex subunit Sst4, sorting receptor for ubiquitinated membrane proteins	+++
SPBC651.05c	*dot2*	ESCRT II complex subunit Dot2	+++
SPBC4B4.06	*vps25*	ESCRT II complex subunit Vps25	+++
SPBC3B9.09	*vps36*	ESCRT II complex subunit Vps36	+++
SPBC215.14c	*vps20*	ESCRT III complex subunit Vps20	+++
SPAC4F8.01	*did4*	ESCRT III complex subunit Did4	+++
SPAC1142.07c	*vps32*	ESCRT III complex subunit Vps32	+++
SPAC17G6.05c	*bro1*	BRO1 domain protein Bro1	+++
SPBC8D2.02c	*vps68*	vacuolar sorting protein Vps68	++
SPAC17A2.06c	*vps8*	CORVET complex WD repeat/ ubiquitin-protein ligase E3 subunit Vps8	+
**Regulation of transcription and translation**			
SPBC25B2.03^a^	NA	zf-C3HC4 type zinc finger	+++
SPBC28F2.02	*mep33*	translation machinery associated protein Mep33	+++
SPBC1861.07	NA	elongin C (predicted)	++
SPBC1718.03^a^	*ker1*	DNA-directed RNA polymerase I complex subunit Ker1	+
**Ubiquitination**			
SPBC31F10.10c^a^	NA	zf-MYND type zinc finger protein	+++
SPBC1105.09	*ubc15*	ubiquitin conjugating enzyme E2 Ubc15	+
SPBC16G5.03	NA	ubiquitin-protein ligase E3	+
**Nucleic acid metabolism**			
SPAC4G9.11c	*cmb1*	cytosine-mismatch binding protein 1	+++
SPAC30D11.07	*nth1*	DNA endonuclease III	++
**Amino acid synthesis and metabolism**			
SPAC22A12.06c	*fsh2*	serine hydrolase-like	++
SPAC4G9.10	*arg3*	ornithine carbamoyltransferase Arg3	+
**Ribosome biogenesis and assembly**			
SPAC3F10.17	*ltv1*	ribosome biogenesis protein Ltv1	++
SPAC3H5.12c	*rpl501*	60S ribosomal protein L5	+
**Histone acetylation and deacetylation**			
SPBC16A3.19	*eaf7*	histone acetyltransferase complex subunit Eaf7	+++
SPAC57A10.14	*sgf11*	SAGA complex subunit Sgf11	++
**Signal transduction**			
SPBC1D7.03^a^	*clg1*	cyclin Clg1 (predicted)	++
SPAC227.15^a^	*reg1*	protein phosphatase regulatory subunit Reg1 (predicted)	++
**Other functions**			
SPBC1778.03c	NA	NADH pyrophosphatase	+++
SPBC660.07	*ntp1*	alpha,alpha-trehalase Ntp1	+++
SPAC11D3.15	NA	5-oxoprolinase (ATP-hydrolizing)	+++
SPAPB1E7.06c	*eme1*	Holliday junction resolvase subunit Eme1	++
SPAC637.10c	*rpn10*	19S proteasome regulatory subunit Rpn10	++
SPAC631.01c	*acp2*	F-actin capping protein beta subunit Acp2	+
SPAC15A10.15	*sgo1*	inner centromere protein, shugoshin Sgo2	+

^a^ The gene is conserved in fungi only.

Other genes are conserved in both yeast and human. +++, strongly resistant, ++, moderately resistant, +, mildly resistant. NA indicates that common gene name is not applicable. *S*. *pombe* gene description was retrieved from the PomBase Database (http://www.pombase.org/).

### Deletion mutants of genes involved in vacuolar protein sorting showed sodium resistance

As described above and shown in Table 1, ten deletion mutants of genes involved in membrane trafficking showed various levels of resistance to sodium chloride. Notably, seven of the ten mutants are subunits of the ESCRT complexes and the two other membrane trafficking mutants, Δ*bro1* and Δ*vps68* are related to the ESCRT function [22, 23]. The remaining Δ*vps8* is a deletion mutant of the gene encoding a subunit of the CORVET (‘class C core vacuole/endosome tethering’) complex that functions in endosome–endosome fusion [24]. On the other hand, some ESCRT mutants such as Δ*sst6* (*vps23*), Δ*vps28* and Δ*vps24* did not show resistance to sodium chloride (Fig 1C).

### ESCRT mutants showed sodium resistance also showed defects in vacuole fusion

**Fig 2:**
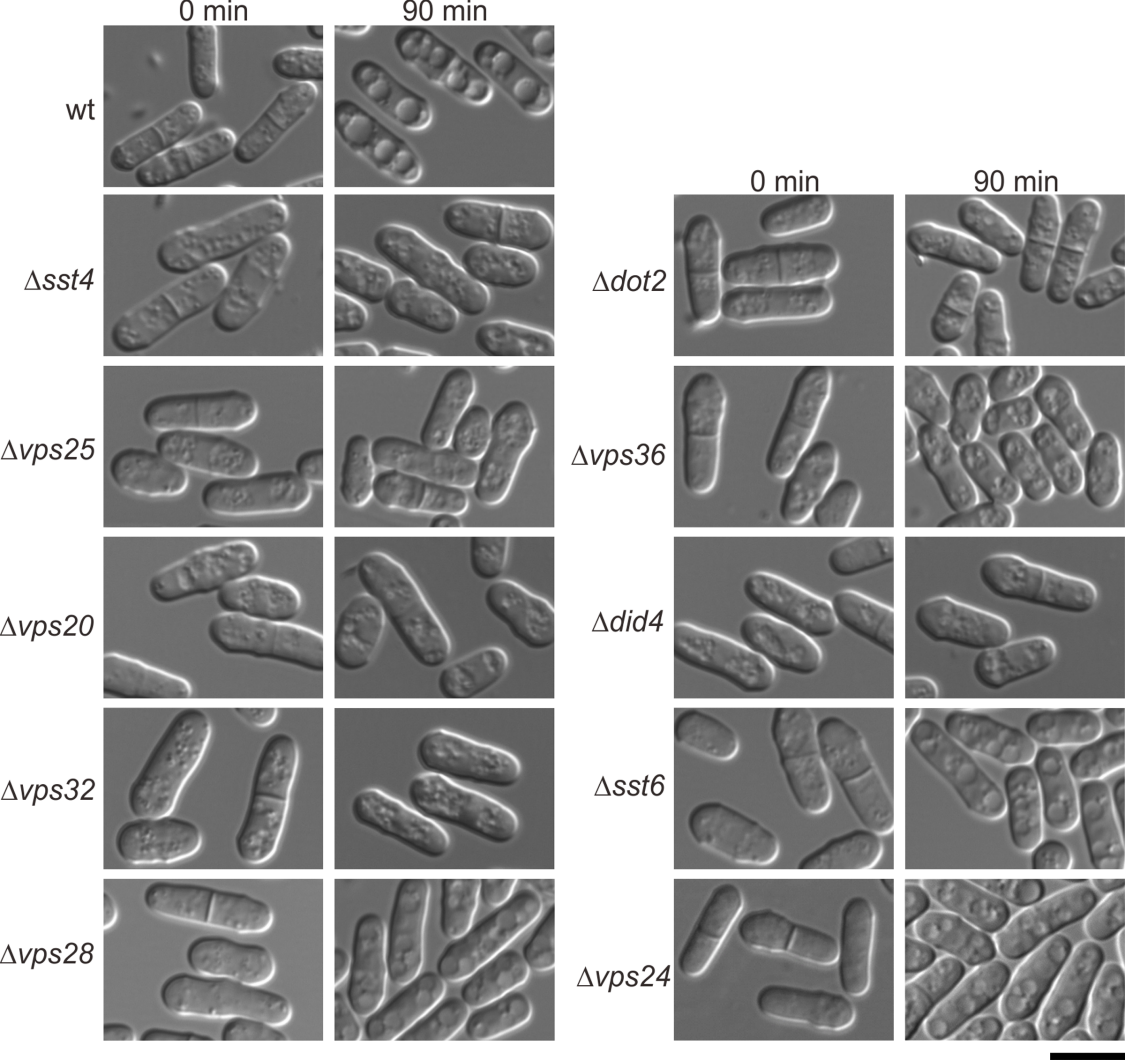
ESCRT mutants that showed sodium resistance were also defective in vacuole fusion. Wild-type cells (wt) and ESCRT mutant cells were grown to log phase in YES medium at 27°C. Then cells were harvested, resuspended in water, and examined by DIC microscopy. Photographs were taken after resuspension in water for 0min and 90min, respectively. Bar: 10μm.

In the *in vivo* screening for deficiencies in vacuolar fragmentation activity of an ordered collection 4881 deletion mutants of *Saccharomyces cerevisiae* (*S*. *cerevisiae*), it has been reported that ESCRT mutants showed vacuolar fragmentation deficiencies [25]. Then we examined vacuole response to low osmolarity in ECSRT mutants as described by Bone *et al*. [26]. DIC microscopy was used to examine vacuole morphology of log-phase cells grown in rich medium and transferred to water for 90 min. As shown in Fig 2, when the wild-type cells were transferred from medium to water, a number of much larger vacuole structures were appeared (Fig 2). In clear contrast, in the ESCRT mutants including Δ*sst4*, Δ*dot2*, Δ*vps25*, Δ*vps36*, Δ*vps20*, Δ*did4* and Δ*vps32* which displayed strong resistance to sodium chloride, vacuoles remained small and numerous (Fig 2), suggesting that these ESCRT complexes are required for vacuole fusion. Notably, ESCRT mutants that were not resistant to sodium chloride, namely Δ*sst6* (*vps23*), Δ*vps28* and Δ*vps24*, did not show detects in vacuole fusion (Fig 2).

### Sensitivity of the ESCRT mutants to various cation salts

**Fig 3:**
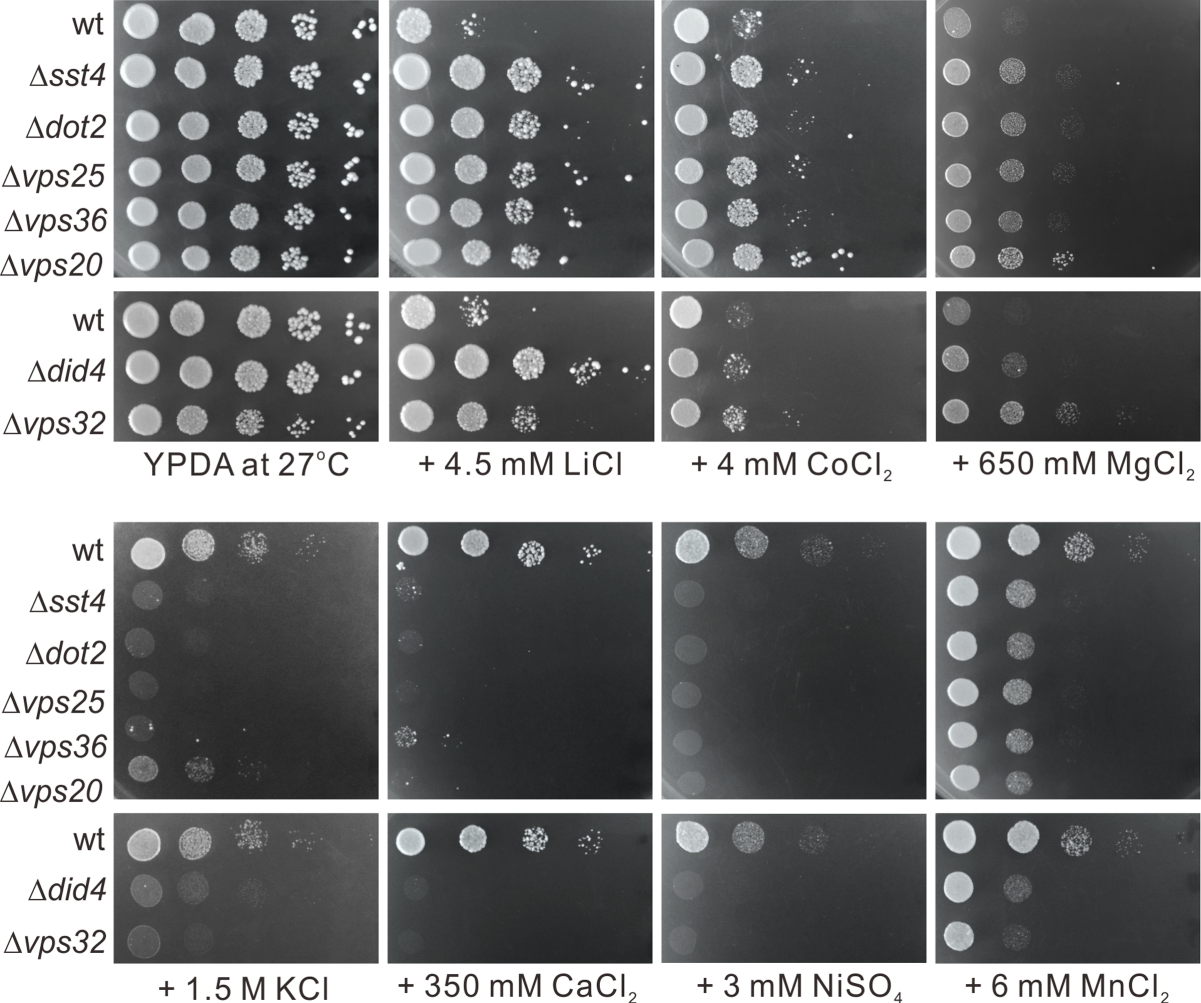
Sensitivity of the ESCRT mutants to various cation salts. Wild-type (wt) cells and ESCRT mutant cells grown at log phase were spotted onto plates containing YPDA or YPDA plus 4.5mM LiCl, 4mM CoCl_2_, 650mM MgCl_2_, 1.5M KCl, 350mM CaCl_2_, 3mM NiSO_4_ and 6 mM MnCl_2_, respectively, and then incubated at 27°C for 4 days.

To further understand the role of the ESCRT pathway in various ion homeostasis, we examined sensitivity of the ESCRT mutants which showed NaCl resistance to various cation salts including LiCl, CoCl_2_, MgCl_2_, KCl, CaCl_2_, NiSO_4_ and MnCl_2_. As shown in Fig 3, the ESCRT mutants which were resistant to NaCl also showed resistance to LiCl, CoCl_2_ and MgCl_2_, however, they showed hypersensitivity to KCl, CaCl_2_, NiSO_4_ and MnCl_2_ (Fig 3).

### Sensitivity of the ESCRT mutants to various drugs

**Fig 4:**
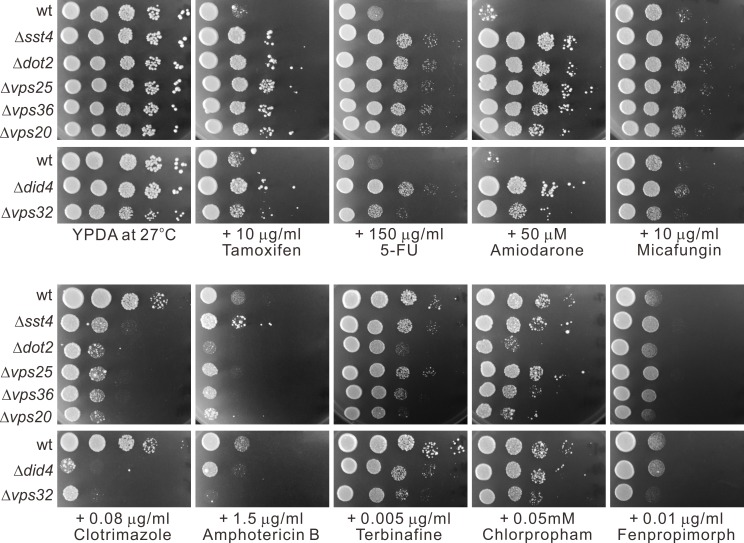
Sensitivity of the ESCRT mutants to various drugs. Wild-type (wt) cells and ESCRT mutant cells grown at log phase were spotted onto each plate as indicated and then incubated at 27°C for 4 days.

The above findings prompted us to examine sensitivity of the ESCRT mutants that showed NaCl resistance to various drugs which are known to inhibit the growth of fission yeast cells. The drugs tested were tamoxifen, 5-FU, amiodarone, micafungin, clotrimazole, amphotericin B, terbinafine, chlorpropham and fenpropimorph. While these ESCRT mutants were equally or more sensitive to most of the drugs tested, they showed resistance to some drugs such as tamoxifen, 5-FU and amiodarone (Fig 4). These results suggest that the ESCRT pathway plays important roles in drug/ion resistance of fission yeast.

### Calcineurin activity and intracellular localization of ion transporters in ESCRT mutants

**Fig 5:**
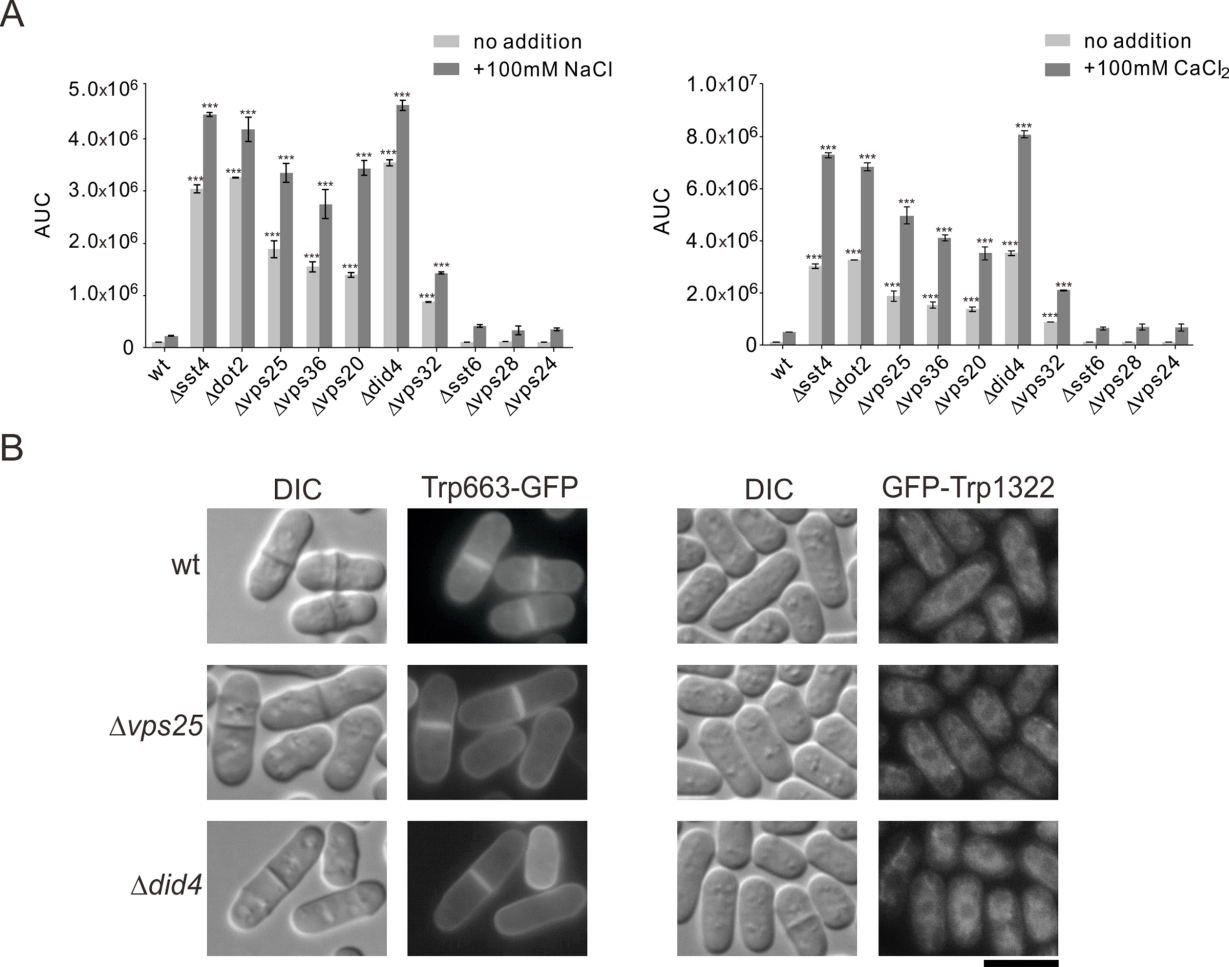
Calcineurin activity and intracellular localization of ion transporters in ESCRT mutants. (A) Real time monitoring of calcineurin activity in ESCRT mutants. Wild-type and ESCRT mutant cells harboring the multicopy plasmid 3×CDRE::luc(2.2) reporter vector were incubated with D-luciferin sodium salt and treated with 100mM NaCl or 100mM CaCl_2_, as indicated. Using a luminometer, light emission levels expressed as relative light units (RLU) were measured per minutes for 3 hours. Graph shows the Area Under Curve (AUC) of 3×CDRE::luc(R2.2) reporter activity untreated or treated with NaCl and CaCl_2_, respectively. The data were averaged from three independent experiments. Error bars, means±SD. ***P<0.001 compared with values from wild-type cells. (B) Intracellular localization of Trp663 and Trp1322 in ESCRT mutants. Wild-type, Δ*vps25* and Δ*did4* cells harboring Trp663-GFP or GFP-Trp1322 were grown to early log phase in EMM plus adenine and uracil media containing 4 μM thiamine, and then were analyzed by fluorescence microscopy. Bar, 10 μm.

To analyze the physiological transport of Ca^2+^ ion through the plasma membrane in ESCRT mutant cells, we monitored calcineurin activity in living cells by using 3×CDRE (calcineurin-dependent response element) fused to destabilized luciferase, because calcineurin activity reflects the level of intracellular Ca^2+^ ion. Our previous results showed that high extracellular NaCl or CaCl_2_ caused an increase in calcineurin activity, but through distinct mechanisms [19]. As shown in Fig 5A, although the ESCRT mutants that showed strong resistance to NaCl were sensitive to CaCl_2_, their calcineurin activities were much higher than that of wild-type cells on basal as well as on both stimulations. Notably, ESCRT mutants that were not resistant to NaCl showed similar calcineurin activities as compared to that of wild-type cells.

The transient receptor potential (TRP) channels have been known to play important roles in regulating cytoplasmic Ca^2+^ [27, 28]. In order to investigate the role of the TRP channels in the ESCRT mutants, we observed the intracellular localization of two TRP channels (Trp1322 and Trp663) in the ESCRT mutants. As shown in Fig 5B, the results showed that localization of these TRP channnels in Δ*did4* and Δ*vps25* cells was similar to those in wild-type cells (Fig 5B). The localization of these TRP channels in the other ESCRT mutants was also similar to those in wild-type cells (data not shown). These results suggest that the ESCRT mutations do not affect the localization of TRP channnels, and NaCl resistance of the ESCRT mutants is not related to intracellular level of Ca^2+^ ion via these two TRP channels.

## Discussion

The multivesicular body (MVB) pathway delivers ubiquitinated membrane proteins into vacuole/lysosomes for degradation. This pathway involves endosomal sorting complexes required for transport (ESCRT). The components of the ESCRT machinery were first identified in budding yeast *S*. *cerevisiae* in the screening of vacuolar protein sorting (*vps*) mutants, which failed to transport proteins into the vacuole [29]. Further genetic and biochemical characterization of the machinery revealed five distinct ESCRT complexes (ESCRT-0, -I, -II, -III and the Vps4 complex) and accessory proteins, each have unique structures and discrete functions [30]. The components of the ESCRT pathway identified in budding yeast *S*. *cerevisiae* are largely conserved in *S*. *pombe* and mammals [30–32].

Bowers *et al*. tested deletion strains for all *vps* class E genes of budding yeast and found that they are sensitive to calcium chloride and lithium chloride [33]. Logg *et al*. showed that deletion strains for several *vps* class E genes including Δ*vps20* and Δ*vps32* are sensitive to sodium chloride [34]. However, as described in this study, in spite of its genetic similarity to budding yeast, we identified many deletion strains of the ESCRT complexes in the screening for sodium chloride resistant mutants in fission yeast *S*. *pombe*. Notably, fission yeast ESCRT mutants showed resistance to LiCl, CoCl_2_ and MgCl_2_, but they showed sensitivity to KCl, CaCl_2_, NiSO_4_ and MnCl_2_. These results suggest that the ESCRT pathway has important roles in ion homeostasis and there are distinct mechanisms in the ESCRT function to regulate homeostasis for various ions. Quite contrary to our results, Luo *et al*. reported that they identified many ESCRT mutants in their screening for nickel-tolerant diploid deletion mutants of budding yeast genes and suggested that the ESCRT pathway is required for the sensitivity of yeast cells to nickel ions [35]. These results suggest that the ESCRT function for ion homeostasis is markedly different in these two yeast species.

Interestingly, ESCRT mutants which were resistant to NaCl showed higher calcineurin activities upon stimulation by NaCl or CaCl_2_ when compared with wild-type cells. As shown in our previous study, activation of calcineurin by high extracellular NaCl is mediated by the Yam8/Cch1 Ca^2+^ channels and is distinct from its activation by high extracellular Ca^2+^ [19]. Our results suggest that defective Ca^2+^ ion regulatory mechanisms in these ESCRT mutants result in high intracelluar Ca^2+^ concentration and CaCl_2_-sensitive phenotype of these mutants.

Ion transporters can be damaged/oxidized in response to ions, which leads to their ubiquitination and degradation through the ESCRT pathway with the collaboration of arrestins [36]. Since we also get some ubiquitination-related mutants in our analysis, ubiquitination process might also be involved in ion response. A possibility is that the difference in sensitivity/resistance to some ions is related to the effect of the ions in the ubiquitination and/or transport through the MVB/vacuoles of the corresponding transporter/channel.

Likewise, the ESCRT mutants showed resistance to some drugs such as 5-FU, tamoxifen and amiodarone while they were equally or more sensitive to other drugs tested. These results suggest that the ESCRT pathway is important for drug metabolism and has important roles in drug sensitivity and metabolism. In addition, these results suggest that the ESCRT function for drug metabolism is also markedly different in these two yeast species and that the ESCRT pathway may be relevant for antifungal drug resistance in pathogenic fungi. Since the ESCRT mutants showed resistance to some ions and drugs while they were equally or more sensitive to other ions or drugs, it is suggested that the ESCRT mutations affect dynamism of plasma membrane proteins and its perturbation causes to increase (or decrease) of import machinery of certain solutes and export machinery of others simultaneously. This may explain resistance of some ions/drugs and sensitivity of other ions/drugs in an ESCRT mutant.

We showed that all the ESCRT mutants which showed sodium resistance also showed defects in vacuole fusion. It has been reported that budding yeast cell contains 2–5 vacuoles of intermediate size during logarithmic growth on rich media, and that hypotonic media promote vacuole fusion whereas hypertonic conditions induce rapid fragmentation [37]. Although there is currently no report regarding ESCRT mutant for the defect in vacuole fusion, it has been reported that budding yeast ESCRT mutants showed vacuolar fragmentation deficiencies under hypertonic conditions [25]. Together with this budding yeast report, our results suggest that the ESCRT pathway is important for the regulation of vacuolar size in various fungi and may be important for the regulation of lysosomal morphology in mammalian cells.

## Conclusions

In this study, we conducted the genome-wide screening of fission yeast haploid nonessential gene deletion mutants to identify mutants resistant to NaCl and have identified 34 NaCl-resistant mutants. Importantly, we showed that some ESCRT mutants were resistant to NaCl as well as drugs such as 5-FU and also were defective in vacuole fusion. In addition, these mutants were sensitive to various salts such as CaCl_2_ and drugs such as clotrimazole. Our results suggest that the ESCRT pathway is relevant for the drug/ion sensitivity and may be involved in regulation of antifungal drug resistance in pathogenic fungi.

## Abbreviations

ESCRT: the endosomal sorting complex required for transport
EMM: Edinburgh minimal medium
ORF: open reading frame
YPD: yeast extract-peptone-dextrose
DIC: differential interference contrast

## References

[1] Lifton RP. Molecular genetics of human blood pressure variation. Science. 1996;272(5262):676–80. . 8614826 10.1126/science.272.5262.676

[2] Serrano R. Salt tolerance in plants and microorganisms: toxicity targets and defense responses. International review of cytology. 1996;165:1–52. . 8900956 10.1016/s0074-7696(08)62219-6

[3] Horie T, Karahara I, Katsuhara M. Salinity tolerance mechanisms in glycophytes: An overview with the central focus on rice plants. Rice. 2012;5(1):11 doi: 10.1186/1939-8433-5-11 ; PubMed Central PMCID: PMC5520831. 27234237 10.1186/1939-8433-5-11 PMC5520831

[4] Palmer BF, Clegg DJ. Electrolyte and Acid-Base Disturbances in Patients with Diabetes Mellitus. The New England journal of medicine. 2015;373(6):548–59. doi: 10.1056/NEJMra1503102 . 26244308 10.1056/NEJMra1503102

[5] Hase CC, Fedorova ND, Galperin MY, Dibrov PA. Sodium ion cycle in bacterial pathogens: evidence from cross-genome comparisons. Microbiology and molecular biology reviews: MMBR. 2001;65(3):353–70, table of contents. doi: 10.1128/MMBR.65.3.353-370.2001 ; PubMed Central PMCID: PMC99031. 11528000 10.1128/MMBR.65.3.353-370.2001 PMC99031

[6] Flowers TJ. Improving crop salt tolerance. Journal of experimental botany. 2004;55(396):307–19. doi: 10.1093/jxb/erh003 . 14718494 10.1093/jxb/erh003

[7] Kennedy PJ, Vashisht AA, Hoe KL, Kim DU, Park HO, Hayles J, et al A genome-wide screen of genes involved in cadmium tolerance in Schizosaccharomyces pombe. Toxicological sciences: an official journal of the Society of Toxicology. 2008;106(1):124–39. doi: 10.1093/toxsci/kfn153 ; PubMed Central PMCID: PMC2563147. 18684775 10.1093/toxsci/kfn153 PMC2563147

[8] Ryuko S, Ma Y, Ma N, Sakaue M, Kuno T. Genome-wide screen reveals novel mechanisms for regulating cobalt uptake and detoxification in fission yeast. Molecular genetics and genomics: MGG. 2012;287(8):651–62. doi: 10.1007/s00438-012-0705-9 . 22806344 10.1007/s00438-012-0705-9

[9] Calvo IA, Gabrielli N, Iglesias-Baena I, Garcia-Santamarina S, Hoe KL, Kim DU, et al Genome-wide screen of genes required for caffeine tolerance in fission yeast. PloS one. 2009;4(8):e6619 doi: 10.1371/journal.pone.0006619 ; PubMed Central PMCID: PMC2720375. 19672306 10.1371/journal.pone.0006619 PMC2720375

[10] Ma Y, Jiang W, Liu Q, Ryuko S, Kuno T. Genome-wide screening for genes associated with FK506 sensitivity in fission yeast. PloS one. 2011;6(8):e23422 doi: 10.1371/journal.pone.0023422 ; PubMed Central PMCID: PMC3151288. 21850271 10.1371/journal.pone.0023422 PMC3151288

[11] Fang Y, Hu L, Zhou X, Jaiseng W, Zhang B, Takami T, et al A genomewide screen in Schizosaccharomyces pombe for genes affecting the sensitivity of antifungal drugs that target ergosterol biosynthesis. Antimicrobial agents and chemotherapy. 2012;56(4):1949–59. doi: 10.1128/AAC.05126-11 ; PubMed Central PMCID: PMC3318361. 22252817 10.1128/AAC.05126-11 PMC3318361

[12] Zhou X, Ma Y, Fang Y, gerile W, Jaiseng W, Yamada Y, et al A genome-wide screening of potential target genes to enhance the antifungal activity of micafungin in Schizosaccharomyces pombe. PloS one. 2013;8(5):e65904 doi: 10.1371/journal.pone.0065904 ; PubMed Central PMCID: PMC3667807. 23738021 10.1371/journal.pone.0065904 PMC3667807

[13] Hu L, Yao F, Ma Y, Liu Q, Chen S, Hayafuji T, et al Genetic evidence for involvement of membrane trafficking in the action of 5-fluorouracil. Fungal genetics and biology: FG & B. 2016;93:17–24. doi: 10.1016/j.fgb.2016.05.007 . 27255861 10.1016/j.fgb.2016.05.007

[14] Zhang X, Fang Y, Jaiseng W, Hu L, Lu Y, Ma Y, et al Characterization of Tamoxifen as an Antifungal Agent Using the Yeast Schizosaccharomyces Pombe Model Organism. The Kobe journal of medical sciences. 2015;61(2):E54–63. . 26628015

[15] Zhang L, Ma N, Liu Q, Ma Y. Genome-wide screening for genes associated with valproic acid sensitivity in fission yeast. PloS one. 2013;8(7):e68738 doi: 10.1371/journal.pone.0068738 ; PubMed Central PMCID: PMC3702616. 23861937 10.1371/journal.pone.0068738 PMC3702616

[16] Kim DU, Hayles J, Kim D, Wood V, Park HO, Won M, et al Analysis of a genome-wide set of gene deletions in the fission yeast Schizosaccharomyces pombe. Nature biotechnology. 2010;28(6):617–23. Epub 2010/05/18. doi: 10.1038/nbt.1628 . 20473289 10.1038/nbt.1628 PMC3962850

[17] Toda T, Dhut S, Superti-Furga G, Gotoh Y, Nishida E, Sugiura R, et al The fission yeast pmk1+ gene encodes a novel mitogen-activated protein kinase homolog which regulates cell integrity and functions coordinately with the protein kinase C pathway. Molecular and cellular biology. 1996;16(12):6752–64. Epub 1996/12/01. ; PubMed Central PMCID: PMC231678. 8943330 10.1128/mcb.16.12.6752 PMC231678

[18] Hoffman CS, Wood V, Fantes PA. An Ancient Yeast for Young Geneticists: A Primer on the Schizosaccharomyces pombe Model System. Genetics. 2015;201(2):403–23. doi: 10.1534/genetics.115.181503 ; PubMed Central PMCID: PMC4596657. 26447128 10.1534/genetics.115.181503 PMC4596657

[19] Deng L, Sugiura R, Takeuchi M, Suzuki M, Ebina H, Takami T, et al Real-time monitoring of calcineurin activity in living cells: evidence for two distinct Ca2+-dependent pathways in fission yeast. Molecular biology of the cell. 2006;17(11):4790–800. doi: 10.1091/mbc.E06-06-0526 ; PubMed Central PMCID: PMC1635391. 16928959 10.1091/mbc.E06-06-0526 PMC1635391

[20] Kita A, Sugiura R, Shoji H, He Y, Deng L, Lu Y, et al Loss of Apm1, the micro1 subunit of the clathrin-associated adaptor-protein-1 complex, causes distinct phenotypes and synthetic lethality with calcineurin deletion in fission yeast. Molecular biology of the cell. 2004;15(6):2920–31. Epub 2004/03/30. doi: 10.1091/mbc.E03-09-0659 ; PubMed Central PMCID: PMC420114. 15047861 10.1091/mbc.E03-09-0659 PMC420114

[21] Fang Y, Sugiura R, Ma Y, Yada-Matsushima T, Umeno H, Kuno T. Cation diffusion facilitator Cis4 is implicated in Golgi membrane trafficking via regulating zinc homeostasis in fission yeast. Molecular biology of the cell. 2008;19(4):1295–303. Epub 2008/01/18. doi: 10.1091/mbc.E07-08-0805 ; PubMed Central PMCID: PMC2291430. 18199682 10.1091/mbc.E07-08-0805 PMC2291430

[22] Kimura Y, Kawawaki J, Kakiyama Y, Shimoda A, Tanaka K. The ESCRT-III adaptor protein Bro1 controls functions of regulator for free ubiquitin chains 1 (Rfu1) in ubiquitin homeostasis. The Journal of biological chemistry. 2014;289(31):21760–9. doi: 10.1074/jbc.M114.550871 ; PubMed Central PMCID: PMC4118134. 24962567 10.1074/jbc.M114.550871 PMC4118134

[23] Schluter C, Lam KK, Brumm J, Wu BW, Saunders M, Stevens TH, et al Global analysis of yeast endosomal transport identifies the vps55/68 sorting complex. Molecular biology of the cell. 2008;19(4):1282–94. doi: 10.1091/mbc.E07-07-0659 ; PubMed Central PMCID: PMC2291407. 18216282 10.1091/mbc.E07-07-0659 PMC2291407

[24] Balderhaar HJ, Ungermann C. CORVET and HOPS tethering complexes—coordinators of endosome and lysosome fusion. Journal of cell science. 2013;126(Pt 6):1307–16. doi: 10.1242/jcs.107805 . 23645161 10.1242/jcs.107805

[25] Michaillat L, Mayer A. Identification of genes affecting vacuole membrane fragmentation in Saccharomyces cerevisiae. PloS one. 2013;8(2):e54160 doi: 10.1371/journal.pone.0054160 ; PubMed Central PMCID: PMC3562189. 23383298 10.1371/journal.pone.0054160 PMC3562189

[26] Bone N, Millar JB, Toda T, Armstrong J. Regulated vacuole fusion and fission in Schizosaccharomyces pombe: an osmotic response dependent on MAP kinases. Current biology: CB. 1998;8(3):135–44. . 9443913 10.1016/s0960-9822(98)00060-8

[27] Montell C. The TRP superfamily of cation channels. Science's STKE: signal transduction knowledge environment. 2005;2005(272):re3 doi: 10.1126/stke.2722005re3 . 15728426 10.1126/stke.2722005re3

[28] Ma Y, Sugiura R, Koike A, Ebina H, Sio SO, Kuno T. Transient receptor potential (TRP) and Cch1-Yam8 channels play key roles in the regulation of cytoplasmic Ca2+ in fission yeast. PloS one. 2011;6(7):e22421 Epub 2011/08/04. doi: 10.1371/journal.pone.0022421 ; PubMed Central PMCID: PMC3139647. 21811607 10.1371/journal.pone.0022421 PMC3139647

[29] Robinson JS, Klionsky DJ, Banta LM, Emr SD. Protein sorting in Saccharomyces cerevisiae: isolation of mutants defective in the delivery and processing of multiple vacuolar hydrolases. Molecular and cellular biology. 1988;8(11):4936–48. ; PubMed Central PMCID: PMC365587. 3062374 10.1128/mcb.8.11.4936-4948.1988 PMC365587

[30] Schmidt O, Teis D. The ESCRT machinery. Current biology: CB. 2012;22(4):R116–20. doi: 10.1016/j.cub.2012.01.028 ; PubMed Central PMCID: PMC3314914. 22361144 10.1016/j.cub.2012.01.028 PMC3314914

[31] Takegawa K, Iwaki T, Fujita Y, Morita T, Hosomi A, Tanaka N. Vesicle-mediated protein transport pathways to the vacuole in Schizosaccharomyces pombe. Cell structure and function. 2003;28(5):399–417. . 14745133 10.1247/csf.28.399

[32] Babst M. A protein's final ESCRT. Traffic. 2005;6(1):2–9. doi: 10.1111/j.1600-0854.2004.00246.x . 15569240 10.1111/j.1600-0854.2004.00246.x

[33] Bowers K, Lottridge J, Helliwell SB, Goldthwaite LM, Luzio JP, Stevens TH. Protein-protein interactions of ESCRT complexes in the yeast Saccharomyces cerevisiae. Traffic. 2004;5(3):194–210. doi: 10.1111/j.1600-0854.2004.00169.x . 15086794 10.1111/j.1600-0854.2004.00169.x

[34] Logg K, Warringer J, Hashemi SH, Kall M, Blomberg A. The sodium pump Ena1p provides mechanistic insight into the salt sensitivity of vacuolar protein sorting mutants. Biochimica et biophysica acta. 2008;1783(6):974–84. doi: 10.1016/j.bbamcr.2008.02.022 . 18395523 10.1016/j.bbamcr.2008.02.022

[35] Luo C, Cao C, Jiang L. The endosomal sorting complex required for transport (ESCRT) is required for the sensitivity of yeast cells to nickel ions in Saccharomyces cerevisiae. FEMS yeast research. 2016;16(3). doi: 10.1093/femsyr/fow028 . 26994103 10.1093/femsyr/fow028

[36] Lin CH, MacGurn JA, Chu T, Stefan CJ, Emr SD. Arrestin-related ubiquitin-ligase adaptors regulate endocytosis and protein turnover at the cell surface. Cell. 2008;135(4):714–25. doi: 10.1016/j.cell.2008.09.025 . 18976803 10.1016/j.cell.2008.09.025

[37] Weisman LS. Yeast vacuole inheritance and dynamics. Annual review of genetics. 2003;37:435–60. doi: 10.1146/annurev.genet.37.050203.103207 . 14616069 10.1146/annurev.genet.37.050203.103207

